# Evaluating swallowing capacity in older adults with dysphagia: high protein, low carbohydrate smoothie formulas versus commercial formula

**DOI:** 10.1186/s12877-025-06126-x

**Published:** 2025-07-02

**Authors:** Pichanun Mongkolsucharitkul, Bonggochpass Pinsawas, Thareerat Watcharachaisoponsiri, Sophida Suta, Sureeporn Pumeiam, Suphawan Ophakas, Apinya Surawit, Sunun Ongard, Phawin Keskool, Poungkaew Thitisakulchai, Phisamai Srichayet, Korapat Mayurasakorn

**Affiliations:** 1 Siriraj Population Health and Nutrition Research Group (SPHERE), Research Group and Research Network Division, Research Department, Faculty of Medicine Siriraj Hospital, Mahidol University, Bangkok, 10700 Thailand; 2 Department of Otolaryngology, Faculty of Medicine Siriraj Hospital, Mahidol University, Bangkok, 10700 Thailand; 3 Department of Rehabilitation Medicine, Faculty of Medicine Siriraj Hospital, Mahidol University, Bangkok, 10700 Thailand; 4 Institute of Food Research and Product Development, Kasetsart University, Bangkok, 10900 Thailand

**Keywords:** Aging, Smoothie drink, Dysphagia, Swallowing capacity

## Abstract

**Background:**

Dysphagia is a common problem in older adults that can lead to nutritional deficiencies. Oral nutritional supplements (ONS) are products used for oral nutrition support as an alternative nutritional therapy, specifically manufactured for the older individuals at risk of dysphagia. This study aimed to develop four high protein (23–34% energy ratio) and low carbohydrate (25–38% energy ratio) smoothie formulas (white sesame (WS) vs. white sesame and low carbohydrate (WSLC) vs. black sesame and low carbohydrate (BSLC) vs. chicken shitake (CS); 1 kcal/ml) for the older people with dysphagia and to assess their effect on swallowing capacity compared to widely used commercial formula (Ensure^®^).

**Methods:**

A cross-sectional study involved 63 participants aged 65 years or over. Subjects were divided into asymptomatic (*n* = 32, aged 72.9 ± 5.66 year) or symptomatic swallowing difficulty (*n* = 31, aged 75.0 ± 6.48 year) groups based on swallowing screening questionnaires. Swallowing capacity was assessed using Fiberoptic Endoscopic Evaluation of Swallowing (FEES), performed by experienced physicians, for three drinks (WS, CS vs. Ensure^®^) in a blinded, randomly crossover sequence.

**Results:**

Spare retention of a food bolus in each formula had been identified in the asymptomatic (47–66%) and symptomatic (61–71%) groups. WS had fewer premature spills than Ensure^®^ in the symptomatic group, but not in the asymptomatic group (5 ± 0.03 vs. 4.7 ± 0.12, *p* < 0.05), while CS had fewer premature spills than Ensure^®^ in both groups.

**Conclusions:**

The findings suggest that smoothie drinks, particularly WS (51–350 centipoise), may offer a viable alternative to commercial formula (1–50 centipoise) for older adults with dysphagia, potentially reducing premature spillage. The findings provide useful preliminary insights into the potential of these formulas to support safer swallowing. Further research, including longer-term studies, would help to more fully explore their nutritional benefits, safety, and applicability across broader populations.

**Trial registration:**

Clinical Trial ID NCT04901182, https//clinicaltrials.gov/ct2/show/NCT04901182 (25/05/2021).

**Supplementary Information:**

The online version contains supplementary material available at 10.1186/s12877-025-06126-x.

## Introduction

The global population is aging, with 771 million people aged 65 years or over in 2022, primarily in developing countries [1]. In 2022, 18.9% of Thais were older individuals, and this is projected to increase to 31.4% by 2042 [2]. Dysphagia, a symptom characterized by difficulty in swallowing, affects at least one-third of older adults diagnosed with neurodegenerative diseases or non-communicable diseases such as stroke, Parkinson’s disease, esophageal dysmotility (e.g. achalasia, or scleroderma), inflammation (e.g. radiation esophagitis), and structural abnormalities (e.g. oral cancer, or peptic strictures) [3–5]. Most seniors with dysphagia eventually experience multiple health issues, leading to a progressive impairment of various organ functions. Muscle mass tends to decline, associated with reductions in strength and musculoskeletal function and coordination [6, 7]. Untreated dysphagia can lead to reduced food intake, suffocation, and aspiration. Dysphagia can also elevate the risk of developing malnutrition and pneumonia [8–10]. Large studies in nursing homes [11] and in a hospital [12] have concluded that individuals with dysphagia face a higher risk of choking, food aspiration, and increased mortality [13]. Managing dysphagia involves addressing underlying causes, utilizing behavioral treatments [14], making environmental modifications [15], practicing swallowing exercises [16], employing compensatory management, and implementing diet modifications [17].

Among Thai individuals aged 60 years or over, malnutrition prevalence ranges from 6 to 10% based on 71 studies [18]. Chaleekrua et al. reported that the prevalence of swallowing problems among healthy Thai community-dwelling older people was 11% [19]. However, official statistics and systematic records on malnutrition in older Thais with swallowing difficulties are lacking. Oral nutritional supplements (ONS) are products used for oral nutrition support as an alternative strategy to treat malnutrition in older individuals with swallowing disorders [20–22]. Swallowing difficulties cause older adults to fear aspiration, leading them to avoid eating. Additionally, the effort required to swallow increases their energy expenditure. Furthermore, when illness occurred, undernourished older adults often experienced complications and longer treatment times [23, 24]. With aging, factors such as tooth loss, impairment of taste sensation, and changes in masticatory muscle strength and integrity become crucial considerations when choosing a nutrition supplement [25, 26]. Interventions for dysphagia encompass both compensatory and rehabilitative methods, aiming to enhance swallow function and mitigate risks such as aspiration, pneumonia, and choking. However, it’s important to note the limited and insufficient evidence regarding the risks and benefits of compensatory approaches [27]. While texture modification following the International Dysphagia Diet Standardisation Initiative (IDDSI) framework is commonly implemented, recent systematic reviews have not definitively demonstrated its effectiveness in reducing dysphagia symptoms or associated complications such as pneumonia or malnutrition. Additionally, a study by Wright [28] comparing the nutritional content of texture-modified diets (TMD) versus normal food found that pureed food was less nutritious than normal food, indicating potential nutritional implications of texture modification [28, 29]. This was primarily due to the challenges of consuming less palatable and energy-dense foods. Regardless of the underlying medical conditions, oral nutrition supports provide protein, fat, energy, and essential micronutrients. These products can be administered orally without contraindications or enterally through a feeding tube [30, 31]. This underscores the notion that good nutrition in an older age with dysphagic risks is linked to healthy aging. Undernourished patients face an increased risk of infections, falls, pressure injuries, and mortality [32]. Maintaining an adequate diet is crucial to meet the needs of older people with comorbidities [33].

Thickened liquids are commonly utilized in dysphagia management to enhance bolus control and reduce the risk of aspiration [34]. However, despite their safety benefits, they can have potential physiological drawbacks. Concerns have been raised about thickeners affecting water binding, especially given the prevalence of dehydration in dysphagia patients. Although they don’t affect water availability, thickeners may hinder medication absorption due to their viscosity. Thickened liquids can delay drug release. Moreover, consuming increasingly viscous fluids may increase feelings of fullness and thirst while also diminishing flavor quality. In Thailand, there is a limited choice of commercially available ONS for dysphagia patients. Many of these are nutritionally sub-optimal, often consisting of high carbohydrate choices like congee or soft-boiled rice. Thailand has officially entered the status of an Aged Society. According to the 6th Thai National Health Examination Survey (2019–2020), the prevalence of diabetes among Thai adults aged 60–69 years was 20.7%, and 21.3% among those aged 70–79 years [35]. Given the high prevalence of diabetes in older adults, the carbohydrate content of dietary interventions requires careful consideration to support glycemic control while maintaining adequate energy and protein intake. The prescription of medical nutrition supplements is relatively low in Thailand due to limitations in public and private insurance reimbursement. Patients with illnesses may have reduced appetites and are often restricted to consuming therapeutic diets for extended periods, putting some patients at risk of inadequate nutritional intake [32]. Pureed foods and moderately thick liquids are helpful in preventing food and fluids from entering the lungs compared to other textures [29, 36]. However, each patient’s ability to tolerate the oral intake of dysphagia diets requires individual evaluation.

The objective of this study was to develop four high-protein texture-modified formulas and to compare their effects on the swallowing capacity of older people with symptomatic dysphagia versus asymptomatic individuals. This comparison was made using fiberoptic endoscopic evaluation of swallowing (FEES) while consuming these formulas and a well-known commercial conventional formula (Ensure^®^). We hypothesized that the different smoothie formulas would have varying effects on the swallowing capacities and signs of swallowing difficulties, with some formulas potentially being easier to swallow or reducing the risk of food suffocation more effectively than others, especially in participants with symptomatic swallowing difficulties.

## Methods

### Modified nutrition-dense smoothie diets

The four developed formulas comprised white sesame soy milk smoothie (WS), white sesame soy milk smoothie (low carbohydrate, WSLC), black sesame soy milk smoothie (low carbohydrate, BSLC), and chicken shitake smoothie (CS) (Supplementary Appendix S1). The composition of energy, carbohydrate, protein, fat, and micronutrients was determined by the Asia Medical and Agricultural Laboratory and Research Center, Bangkok, Thailand, according to the AOAC standard protocol [37]. The four smoothies developed for use in this study provide a normocaloric (1.0-1.1 kcal/ml), hypoglycedic (25–38%), hyperproteic (24–28%) nutritional composition (Table 1). Ensure^®^, a widely used commercial formula (Abbott Nutrition, USA) served as a control. Ensure^®^ was prepared from vanilla powdered formula with a standard 1 kcal/ml recipe according to the manufacturer’s instructions, contained 54% carbohydrate, 15% protein, and 29% fat. Detailed physical properties of the smoothies, including color, pH, and viscosity, can be found in Table 2. The color values were measured using color measurement spectrophotometer (Model Color Quest XE, Hunter lab, USA) in CIE-color system (L*, a*, b*). The color parameters: L* represents lightness from black to white (0 to 100); a* represents redness from green (-) to red (+); and b* represents yellowness from blue (-) to yellow (+). The acidic/basic values were measured using a pH meter (Model SevenCompact, Mettler-Toledo, Switzerland). The viscosity values were measured using a Coaxial spindle CCT-40, Rheometer (Model RST-CC TouchTM, Brookfield Engineering Laboratories, Inc, USA). All formulas had a nectar-like texture (51–350 cP) except Ensure^®^, which was classified as a thin liquid (1–50 cP) based on the National Dysphagia Diet (NDD) criteria [38]. The smoothies were prepared in one batch to maintain homogeneity. For testing, 5 ml of each smoothie and Ensure^®^ were placed in tasting cups and blind-labeled with random three-digit codes.

**Table 1:** Composition of nutritionally dense smoothies and widely used commercial formula per 100 g

Nutrient compositions	WS^a^	WSLC^a^	BSLC^a^	CS^a^	Ensure^®^ (control)^b^
Energy (kcal)	105	103	106	107	100
Carbohydrate (g, %)	10.1, 38	7.1, 28	6.8, 25	9.6, 36	13.4, 54
Protein (g, %)	6.3, 24	7.3, 28	6.6, 25	7.4, 28	3.7, 15
Fat (g, %)	4.3, 37	5.1, 44	5.9, 50	4.3, 36	3.3, 29
Saturated fat (g)	1.9	2.1	2.1	2.6	0.3
Cholesterol (mg)	5.7	15.9	17.3	18.5	N/A
Dietary fiber (g)	0.6	0.5	1.5	0.8	1
Sugar (g)	4.3	3.2	4.8	2	N/A
Sodium (mg)	53	64	46.9	143	83.7
Calcium (mg)	24	25	64.2	41.9	104.7
Iron (mg)	0.8	0.8	1.1	0.9	0.6

Abbreviations: BSLC, black sesame soy milk smoothie (low carbohydrate); CS, chicken shitake smoothie; g, gram; kcal, kilocalorie; mg, milligram; N/A, not available; WS, white sesame soy milk smoothie; WSLC, white sesame soy milk smoothie (low carbohydrate)

^a^Analysis from Asia Medical and Agricultural Laboratory and Research Center Co., Ltd

^b^Data from Abbott Nutrition (prepared from Ensure^®^ powder 23.3 g in water 84.8 g)

**Table 2:** Physical properties of test diets

Test diets	Color			pH	Viscosity (Centipoise)
	L*	a*	b*		
**WS**	64.58 ± 0.07	6.88 ± 0.10	26.78 ± 0.27	6.04 ± 0.01	193.99 ± 17.59
**WSLC**	66.44 ± 0.41	6.93 ± 0.02	26.93 ± 0.21	6.17 ± 0.00	154.82 ± 9.73
**BSLC**	56.82 ± 0.40	3.02 ± 0.02	15.29 ± 0.14	6.04 ± 0.01	214.47 ± 29.18
**CS**	58.73 ± 0.18	6.23 ± 0.09	25.07 ± 0.16	6.11 ± 0.02	80.85 ± 0.07
**Ensure** ^®^ **(control)**	80.89 ± 0.05	0.28 ± 0.01	15.54 ± 0.01	5.97 ± 0.00	6.20 ± 0.10

Abbreviations: BSLC, black sesame soy milk smoothie (low carbohydrate); CS, chicken shitake smoothie; WS, white sesame soy milk smoothie; WSLC, white sesame soy milk smoothie (low carbohydrate)

The L* value indicates lightness (0 = black, 100 = white), a* measures red/green (positive = red, negative = green), and b* measures yellow/blue (positive = yellow, negative = blue)

### Study participants

Adults aged 65 years or over were recruited through social media advertisements and posters at Siriraj Hospital, Mahidol University, Bangkok, Thailand. Inclusion criteria required the ability to understand in the Thai language and to follow physicians’ instructions during a swallowing test. Participants with severe dysphagia (defined as difficulty swallowing that prevents patients from eating and drinking enough, even with treatment to allow safe swallowing), a history of tube feeding, or a history of facial bone or skull surgery were excluded. Additionally, individuals in palliative care, bedridden, or unable to provide informed consent were also excluded. Participants were separated into two groups by using screening questionnaires to ask whether they have experienced certain symptoms more than once per week. The time period considered for these symptoms was the previous month. This allows for the assessment of both recent and recurring swallowing difficulties (Supplementary Appendix S2). Participants without a history of swallowing difficulties, coughing, or choking when eating or drinking were assigned to an asymptomatic swallowing difficulty (ASD) group. Participants with symptomatic swallowing difficulties or with a history mentioned above in the ASD group were assigned to a symptomatic swallowing difficulty group (SSD). At least 30 patients in each group were the sample size that was needed to provide statistical significance to the results. This was based on the suggestion that for estimating the sample size for a pilot trial, a general flat rule to “use at least 30 subjects or greater to estimate a parameter” can be applied [39, 40]. Informed consent was obtained, and the study was approved by the Siriraj Institutional Review Board (COA no. Si 010/2018). The demographic data of participants, including sex, age, and underlying diseases, were collected from the screening questionnaire. The screening questionnaire also included patients’ self-reports about the types of foods that they were able to eat and a history of swallowing problems occurring more than once per week. The study was conducted as a cross-sectional study. The random three-digit codes were used for blind labeling diets and the diets being provided to participants by research staff. This was done to ensure that participants and physicians remained unaware of the specific formulas provided.

### Sensory evaluation

Sensory assessments were conducted using a 9-point hedonic scale to determine the acceptability of the four formulas compared to Ensure^®^ (Figure S1, Supplementary Appendix S3). Sensory attributes evaluated included characteristic, color, smell, taste, viscosity, homogeneity, swallowing, and overall satisfaction [41]. Sensory evaluations were performed consecutively with 5-minute intervals and were followed by face-to-face interviews with research staff. Participants were provided with water to clean their mouths between samples, and the order of formula presentation was randomized crossover for each participant, without explanation of formula attributes.

### Swallowing test

Swallowing capacity, as assessed by FEES, refers to the functional ability to safely and efficiently manage and transport food or liquids through the oropharynx to the esophagus, without signs of aspiration or penetration. This includes observing the coordination of swallowing structures and the effectiveness of bolus clearance [42]. The FEES was performed by two well-trained and experienced physicians (an otolaryngologist and a physiatrist) [42]. Both physicians hold the necessary expertise and certification to safely and effectively conduct FEES examinations, prioritizing patient safety and well-being. To ensure consistency and reliability in assessments, both physicians adhered strictly to the same standardized protocol and established guidelines. During FEES, a flexible laryngoscope with a 4.0 mm diameter distal chip was passed transnasally, with the use of topical anesthetics (3% ephedrine and 4% lidocaine) [43] when the participants sat upright. The tip of the endoscope was placed within the oropharynx beneath the soft palate to visualize the pharynx and larynx before and after all liquid swallows. Two of the developed smoothies, WS (194 cP) and CS (81 cP), were selected for the swallowing test. Participants received three trials of 5-ml food boluses (WS, CS, and Ensure^®^) in a randomized sequence via a spoon by research staff, with instructions to swallow once on cue (Figure S1). The endoscope was then placed above the vocal cords to visualize gross aspiration into the trachea. After each trial, participants were allowed to drink 30 ml of green-dyed water to minimize food residue. The examination was halted if aspirated liquid was detected in any trial. A physician assessed the severity of swallowing disorders, and gastrointestinal complications (nausea, vomiting, abdominal distension, and gastric residue) and adverse events were recorded to evaluate the safety of the trials. Food residue was evaluated based on FEES findings, considering the presence or absence of post-swallow residue [44]. In our clinical practice (at Ear Nose Throat clinic, Department of Otorhiolaryngology, Siriraj Hospital), we used a five-point scoring system suggested by Zacharek et al. [45] and Schindler et al. [46] to assess each parameter [47]. The scores were weighed toward three anatomical regions: premature spillage when the bolus leaks or falls into the hypopharynx before swallowing, retention of the bolus and/or secretion, and entrance of the bolus into the larynx or trachea. The physician rated the food residue as a percent of the space filled by assigning 5 scores based on the perception of the amount of residue compared to the total amount of bolus swallowed (Supplementary Appendix S4) [48].

### Statistical analysis

Statistical analyses were performed using STATA version 15.0 (Stata Corporation, College Station, TX, U.S.). Median ratings of taste and appearance for the test diets (WS, WSLC, BSLC, CS and Ensure^®^) were compared using nonparametric Kruskal-Wallis analysis by ranks. When significant differences were observed (*p* ≤ 0.05), Mann–Whitney U tests were performed on different combinations of the five test diets to determine the individual differences between them, and the *p*-values were adjusted for multiple comparisons using Bonferroni adjustment. Associations between scores and categorical variables were assessed using chi-square or Fisher’s exact tests. Severity scores of swallowing disorders were compared using Friedman test and pairwise Wilcoxon signed rank test.

## Results

### Baseline characteristics

The characteristics of the participants are presented in Table 3. A total of 65 participants, with a mean age of 75.0 ± 6.48 years and 54% females, participated in the sensory evaluation. The primary underlying diseases among participants were hypertension, dyslipidemia, and diabetes mellitus. The swallowing screening identified solid foods, dry foods, and viscous foods as the top three types of foods causing difficulty for participants. The participants reported the top three symptoms of swallowing disorders as stuck food in the throat, choking on food, and repeated swallowing. Two participants participated solely in the sensory test due to personal reasons and did not attend the swallowing test. Of the 63 participants in the swallowing test, 32 were classified in the ASD group, and 31 in the SSD group based on their self-report on the screening questionnaire. The ASD group included two individuals with previous strokes, while the SSD group comprised two participants with neurological histories and eight with head and neck cancer.

**Table 3:** Baseline characteristics of participants who participated in the sensory and swallowing test

Demographic data	All participants (*N* = 65)	Participants based on their self-report swallowing disorder (*N* = 63)	
		ASD (*n* = 32)	SSD (*n* = 31)
**Age (years)**, M**ean (SD)**	75.0 ± 6.48	72.9 ± 5.66	75.0 ± 6.48
**Female**,** n (%)**	35 (53.8)	22 (68.8)	11 (35.5)
**Underlying diseases**,** n (%)**^**a**^			
Hypertension	33 (56.9)	13 (46.4)	19 (65.5)
Dyslipidemia	33 (56.9)	17 (60.7)	16 (55.2)
Diabetes Mellitus	15 (25.9)	11 (39.3)	4 (13.8)
Cancer	8 (13.8)	0 (0.0)	8 (27.6)
Stroke	4 (6.9)	2 (7.1)	2 (6.9)
Gastritis	1 (1.7)	0 (0.0)	1 (3.4)
Others	28 (48.3)	10 (35.7)	17 (58.6)
None	7 (10.8)	4 (12.5)	2 (6.5)
**Swallowing screening**,** n (%)**			
Types of foods, which patients were able to eat^b^			
Clear liquid foods	60 (92.3)	32 (100.0)	26 (83.9)
Full-liquid foods	61 (93.8)	32 (100.0)	27 (87.1)
Soft foods	59 (90.8)	32 (100.0)	25 (80.6)
Semi-solid foods	60 (92.3)	32 (100.0)	26 (83.9)
Viscous foods	58 (89.2)	32 (100.0)	24 (77.4)
Solid foods	57 (87.7)	32 (100.0)	23 (74.2)
Dry foods	58 (89.2)	32 (100.0)	24 (77.4)
History of swallowing disorders, yes (> 1 time/week)^c^	31 (47.7)	0 (0.0)	31 (100.0)
Food stuck in throat	23 (74.2)	0 (0.0)	23 (74.2)
Choking on food	15 (48.4)	0 (0.0)	15 (48.4)
Painful swallowing	3 (9.7)	0 (0.0)	3 (9.7)
Throat irritation	7 (22.6)	0 (0.0)	7 (22.6)
Nasal regurgitation	2 (6.5)	0 (0.0)	2 (6.5)
Repeated swallowing	13 (41.9)	0 (0.0)	13 (41.9)
Not able to eat dry foods or liquid foods	7 (22.6)	0 (0.0)	7 (22.6)

Note: Continuous variables are presented as mean. Categorical variables are presented as count (percentage of participants in each group)

Abbreviations: ASD, asymptomatic swallowing difficulty; SD, standard deviation; SSD, symptomatic swallowing difficulty

^a^ participant may have more than one underlying disease

^b^ participants were able to eat more than one kind of food

^c^ participants may have more than one swallowing disorder condition

### Sensory acceptability

**Fig. 1:**
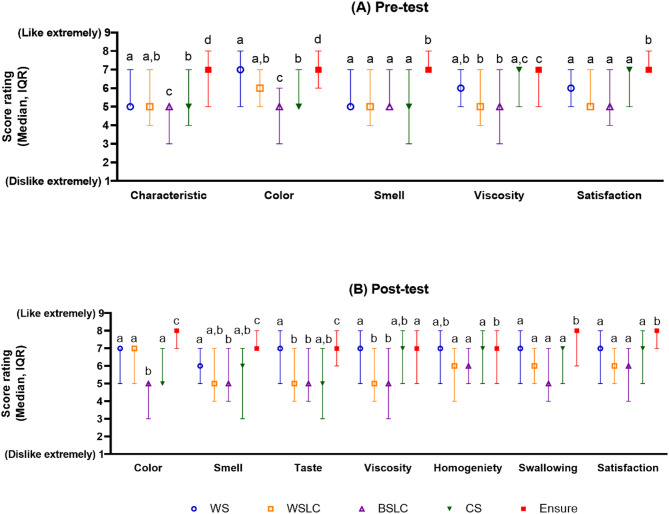
Participants evaluation of key properties of test diets on a 9-point hedonic scale. Panel (**A**) Pre-test: participants evaluation before tasting, Panel (**B**) Pre-test: participants evaluation after tasting. ^*^Significant difference between test diets using the nonparametric Kruskal-Wallis method (*p* < 0.05). Different superscript letters (a, b,c, d) indicate significant differences between test diets using Mann-Whitney comparisons (Bonferroni adjusted) (*p* < 0.05). Abbreviations: BSLC, black sesame soy milk smoothie (low carbohydrate); CS, chicken shitake smoothie; IQR, Interquartile range; WS, white sesame soy milk smoothie; WSLC, white sesame soy milk smoothie (low carbohydrate)

Sensory evaluation of key properties of test diets using a 9-point hedonic scale indicated that the sensory rating of test diets ranged from neutral to ‘like very much’ (Fig. 1, Table S1). Participants rated Ensure^®^ significantly higher in characteristic, color, smell, taste, swallowing, and overall satisfaction compared to modified nutritionally dense smoothie diets. Participants rated Ensure^®^ higher than BSLC and WSLC in terms of viscosity. Similarly, when evaluating homogeneity, participants rated Ensure^®^ higher than BSLC, WSLC, and CS. Color preference leaned toward WS and WSLC over BSLC and CS. The median smell rating score in WS and CS was higher after taste. Based on 9-point hedonic scale, participants moderately favored the viscosity of WS, CS, and Ensure^®^ both before and after testing. In terms of taste, the participants moderately liked Ensure^®^ and WS. Participants moderately liked the homogeneity and swallowing of WS, CS, and Ensure^®^. Overall satisfaction tended to increase after tasting across all formulations.

### Swallowing disorders detected by FEES

**Fig. 2:**
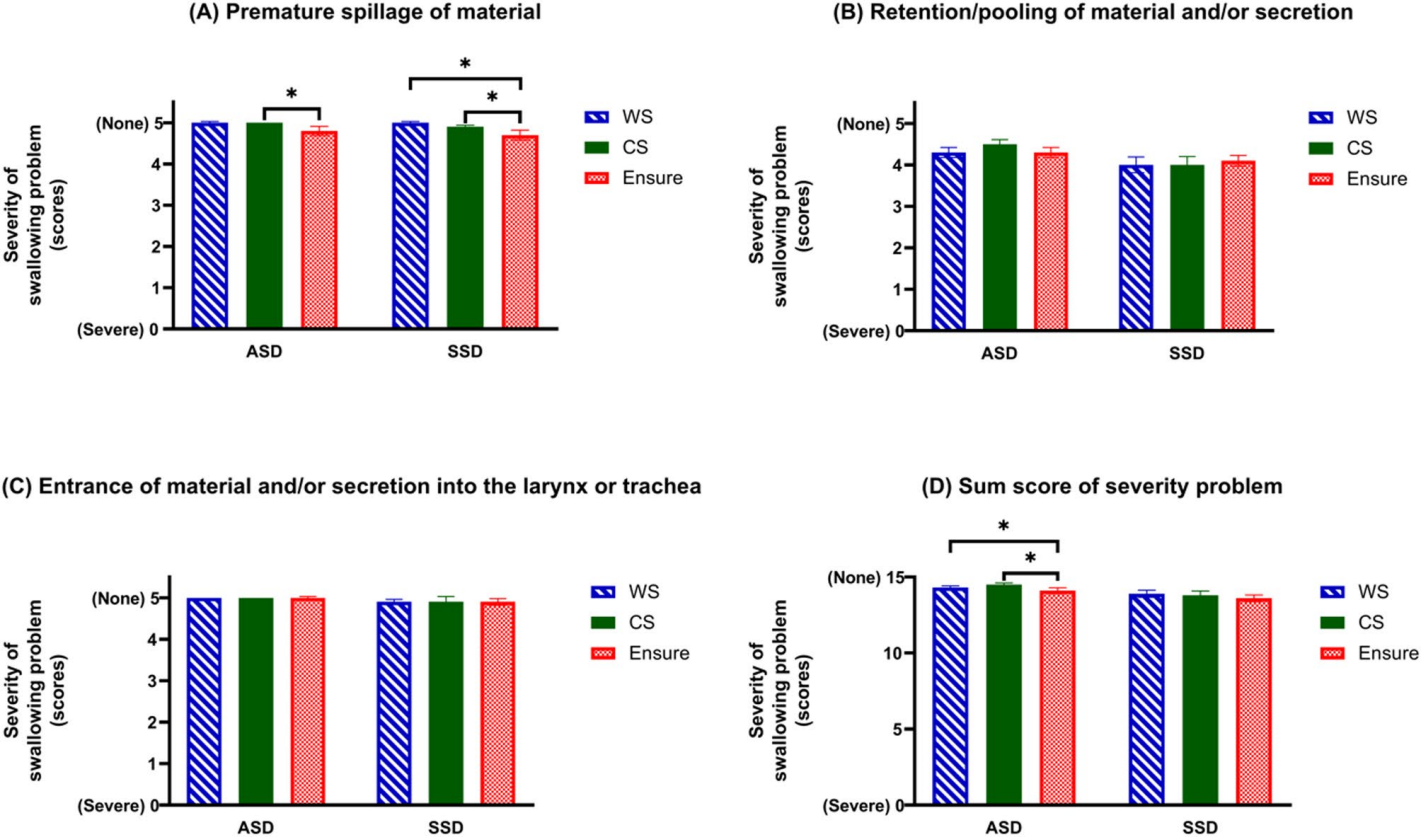
Severity of swallowing disorders in test diets detected by FEES evaluation. Panel (**A**) premature spillage when the bolus leaks or falls into the hypopharynx before swallowing, Panel (**B**) retention of the bolus and/or secretion, Panel (**C**) entrance of the bolus into the larynx or trachea, Panel (**D**) sum score of severity problem. ^*^Significant difference between test diets using Friedman test and pairwise Wilcoxon signed rank test (*p* < 0.05). Higher scores mean less severe swallowing disorders. Abbreviations: ASD, asymptomatic swallowing difficulty; BSLC, black sesame soy milk smoothie (low carbohydrate); CS, chicken shitake smoothie; SSD, symptomatic swallowing difficulty; WS, white sesame soy milk smoothie; WSLC, white sesame soy milk smoothie (low carbohydrate)

Two physicians assessed four swallowing abnormalities using FEES. No occurrences of complications or adverse events were observed. Most participants in the ASD group experienced bolus retention when swallowed WS (66%) and Ensure^®^ (59%). In the SSD group, most participants experienced bolus retention when swallowing all formulas, especially Ensure^®^ (71%). Laryngeal penetration was observed in the SSD group during the swallowing of WS (13%), CS (6.5%), and Ensure^®^ (13%) (Table S2). The proportion of participants in each severity level of swallowing disorders detected by FEES is shown in Table S3. Initially, we hypothesized that in the ASD group, FEES scores would be normal, and that smoothies with the highest viscosity would provide better FEES scores. Additionally, the different smoothie formulas would have varying effects on the swallowing capacities and signs of swallowing difficulties, with some formulas potentially being easier to swallow or reducing the risk of food suffocation, especially in participants with SSD. 16% and 23% of the ASD and SSD groups, respectively, had mild to marked premature spillage when they swallowed Ensure^®^. 53% of participants in the ASD group and 42% of participants in the SSD group were able to swallow CS without retaining or secreting any material. Almost 10% (*n* = 3) of the SSD group had materials leakage into the larynx above the trachea when swallowed Ensure^®^. This was considered laryngeal penetration. Notably, no significant associations were found between severity levels and specific dietary formulations. When comparing between the formulas (Fig. 2 and Table S4), it was found that the sum score of severity problem of the ASD showed the significance differences between WS and Ensure^®^ (*p* = 0.008), and CS and Ensure^®^ (*p* = 0.033). The premature spillage of material score of the ASD group showed a significant difference (*p* = 0.034) between CS and Ensure^®^. While the premature spillage of material score of the SSD group showed the significant differences between WS and Ensure^®^ (*p* = 0.014), and CS and Ensure^®^ (*p* = 0.023).

### Subjective perception of dysphagia

The subjective perception of swallowing difficulty, as reported by patients in the screening questionnaire before FEES evaluation, was determined. Notably, 50% (32/63 participants) of the participants were classified in the ASD group when reporting no history of swallowing difficulties, coughing, or choking when eating or drinking. However, upon FEES assessment, 37% (12/32 participants) of the ASD group exhibited signs of the disorder (FEES score < 15) across all diet formulas (Table S3). Most of the signs found in this group were bolus retention with mild severity. In contrast, 23% (7/31 participants) of the SSD group, reporting a history of swallowing disorders, did not exhibit signs of swallowing disorders (FEES score = 15) across all diet formulas when detected by FEES (Table S3).

## Discussion

In this study, we aimed to evaluate the effects of modified nutrition-dense smoothie diets on swallowing capacities and signs of swallowing difficulties in older adults. We hypothesized that these smoothie formulas, with their higher protein content (6.3–7.4 g/100 g diet) compared to Ensure^®^ (3.7 g/100 g diet), would be easier to swallow and potentially reduce the risk of food suffocation, especially in participants with SSD. Additionally, we hypothesized that the ASD group would show normal FEES scores and that smoothies with higher viscosity would provide better FEES scores. By comparing the effects of these high-protein, texture-modified formulas on the swallowing capacity of older adults, we aimed to identify formulas that could improve swallowing ease and safety. This research contributes to the development of oral nutrition supplement that can better meet the nutritional needs of older adults with swallowing difficulties. Modified nutrition dense smoothie diets were formulated as ready-to-eat meal supplement derived from natural ingredients, containing essential nutrients tailored for the older individuals. Older individuals necessitate higher dietary protein intake, up to 1.2 g/kg body weight/day, to counteract sarcopenia and maintain musculoskeletal health [49]. Unfortunately, meeting these protein requirements poses challenges for many older adults, often attributed to factors such as tooth loss, which can impede the eating process. Therefore, a nutrition dense diet supplement with texture modification has the potential to enhance overall food intake. This study evaluated these modified nutrition-dense smoothie diets and Ensure^®^ using 65 older adults attending a swallowing clinic at the Siriraj hospital. Key findings indicated that solid foods, dry foods, and viscous foods were identified as the top three categories causing difficulty for participants. The predominant symptoms of swallowing disorders reported here were food getting stuck in the throat, choking on food, and repeated swallowing-early indicators of swallowing disorders in the older persons. A modified barium swallow study found that oropharyngeal swallowing physiology evolves with age in healthy adults. Adults aged 60 years and older exhibited significantly worse pharyngeal total scores than younger adults (*p* < 0.01) [50]. Additionally, other signs of dysphagia include coughing, nasal passage of food or liquid, and chewing difficulties. An epidemiological study demonstrated variable dysphagia prevalence in the older population across different medical conditions, ranging from 50 to 80% in nursing home residents with Alzheimer’s or Parkinson’s disease to 30–40% in the community [15, 17, 51]. These findings underscore the significance of addressing swallowing difficulties in older adults, particularly those with neurodegenerative conditions and multiple treatment modalities plays a crucial role in the management. Sensory factors, particularly food temperature, influenced participants’ preferences. In addition, the delicious taste emerged as a pivotal factor influencing individual food preferences more than other sensory aspects [52]. In this study, participants assessed various sensory attributes, revealing that that Ensure^®^ received the highest scores, likely owing to its familiarity. According to our preliminary questionnaire, 42% of participants had previously used oral nutritional supplements formulated specifically for older adults. Of those, 56% had used Ensure^®^, which suggests that familiarity with the product may have influenced their preference. Moreover, Ensure^®^ is a major brand of nutritional supplements targeted at adults and is widely used in Thai hospitals. However, the modified nutrition-dense smoothie diets were well-accepted by the older individuals, with the median scores ranging from neutral to ‘like very much’ on the 9-point hedonic scale. The sensory perception results can have significant clinical applications. The four smoothie formulas presented in this study offer an alternative nutritional option for patients with dysphagia. While Ensure^®^ is a globally recognized and well-researched brand with established nutritional benefits, the smoothies were designed to address specific limitations related to cost, patient acceptability, and adherence. In Thailand and many other countries, Ensure^®^ is not reimbursable by healthcare systems, making it less accessible for some patients. Additionally, the perception of such supplements being associated with illness can impact patient compliance. By contrast, many traditional dysphagia diets rely on thickeners, which, although effective in improving swallowing safety, often result in suboptimal taste. The smoothies aim to provide a more palatable option that could enhance patient satisfaction and adherence, though further comparative studies are needed to evaluate their nutritional benefits against established products like Ensure^®^. The diet for managing dysphagia necessitates incorporating texture-modified foods to facilitate safe and efficient swallowing, alongside sensory stimulation via transient receptor potential (TRP) channel activation to enhance the swallowing reflex [53]. Patients receiving texture-modified diets frequently fail to meet their nutritional needs. It becomes challenging to make texture-modified diets look appetizing, which is essential for achieving the best possible outcomes and enhancing patients’ experience [54]. The four smoothie formulas developed in this study offer a palatable alternative, potentially improving patient compliance and nutritional intake. These formulas were designed to align with the nutritional requirements of older adults with dysphagia, emphasizing high protein content [55]. While Thailand does not have specific regulatory definitions for low carbohydrate foods, the Diabetes Association of Thailand recommends a low carbohydrate eating pattern, where carbohydrates constitute approximately 10–25% of total energy intake [56]. This aligns with international definitions, which characterize low carbohydrate diets as providing 50–130 g of carbohydrates per day, typically corresponding to less than 26% of total energy intake [57, 58]. In this study, we defined “low carbohydrate” as formulas providing less than 30% of total energy from carbohydrates. This threshold is supported by evidence-based recommendations and has been widely used in previous studies as a practical definition for low carbohydrate diets [59]. While the benefits of low carbohydrate diets have been well-documented in specific populations, including individuals with diabetes [60], further studies are needed to confirm these benefits in older adults with dysphagia. Notably, the favorable taste and texture profiles observed in this study suggest that these formulas may enhance eating experience and acceptance, which are critical for improving quality of life in this population.

For the swallowing test, a physician assessed participant for four signs of swallowing difficulty (retention of bolus, nasopharyngeal regurgitation, laryngeal penetration, aspiration). In the ASD group, signs of bolus retention were observed across all three test diets, with mild retention being particularly notable when swallowing WS and Ensure^®^. Two important insights can be derived from this information. This suggests that approximately 60% of normal older individuals may experience early-stage swallowing problems that are not yet causing noticeable issues. Participants in our study may have had diminished sensory perception, which affects their ability to detect bolus retention. Additionally, the mean age across all three groups was comparable, which supports the notion that these findings may not be exclusively attributed to age-related changes but rather to individual differences in sensory and motor function. These may be indicative of presbyphagia, associated with anatomical and functional changes in swallowing physiology during aging in healthy older adults, often undiagnosed as dysphagia [61]. Older individuals with presbyphagia are at a higher risk of developing more severe dysphagia in the future, and silent aspiration may occur if they are unaware of the existence of dysphagia [50]. Studies have shown that while some residue is common, the critical issue is the risk of aspiration. Aspiration occurs when material enters the airway below the vocal folds, which can lead to pneumonia and other respiratory issues. The key is whether the retained material is cleared adequately with subsequent swallows or if it poses a risk of aspiration. For example, Marik and Kaplan noted that while residue is a concern, it becomes problematic primarily when it leads to aspiration pneumonia, a significant risk for older adult patients and those with compromised immune systems [62]. Additionally, the Royal College of Speech and Language Therapists (RCSLT) highlights that management strategies should focus on minimizing aspiration risks rather than completely eliminating residue, which may be unrealistic for many patients with dysphagia [63]. Effective compensatory techniques, such as modifying swallowing posture or texture of food, can help manage residue without necessarily increasing adverse outcomes.

The FEES exam, a gold standard method for evaluating the early stage of swallowing disorders, prompted physicians to advise older individuals with presbyphagia to perform tongue-strengthening exercises and select appropriate food textures to enhance their swallowing. Secondly, sticky foods tend to be problematic and increase the risk of choking and residue. The tongue, which gradually weakens with aging, playing a critical role in pushing food boluses downward toward the pharynx during the swallowing process. The maximum tongue strength, which diminishes with aging, affects the food in the vallecula after swallowing. However, the incidence of mild to marked retention of bolus when swallowed Ensure^®^ (71%) was higher than when swallowed WS (61%) and CS (55%). In our study, while we did not directly assess the stickiness of the different formulas, we observed that WS and CS, despite their higher viscosity compared to Ensure^®^, did not appear to increase residue risk.

The severity of swallowing disorders detected by FEES evaluation was categorized into three signs. The first sign was premature spillage of material, assessing food spillage before swallowing. This occurs during feeding and before the pharyngeal swallow condition, rendering an individual unable to prevent liquid food from flowing down the throat. The ASD group demonstrated proficiency in preventing premature spillage, while the SSD group exhibited a significant difference (*p* < 0.05) between swallowing WS and Ensure^®^. In the SSD group, premature spillage of material occurred more frequently when swallowing Ensure^®^. Premature spillage is a marker of aspiration risk, which also depends on the amount of food consumed. Therefore, it is not practical to use a high volume of food content for safety assessments. However, if the food content in a real-life scenario is higher, we are likely to see a significant difference in outcomes. Even if the results for penetration/aspiration do not differ significantly, the presence of premature spillage indicates a potential risk for aspiration. This finding supports the need for careful consideration and management in clinical practice. The risk of food aspiration may increase due to the premature spilling of food into the oropharynx and its subsequent entry into the unprotected laryngeal opening before the pharyngeal phase of swallowing is triggered [64]. It does increase the risk of pneumonia. Aging-related decreases in sensory-motor physiology, including reduced tongue strength and neurological function, may limit the ability of older individuals to hold food boluses before swallowing, particularly with low-viscosity liquid foods. Modified nutrition-dense smoothie diets were classified as nectar-like texture (51–350 cP) according to NDD criteria [38] and extremely thick drinks or pureed foods according to IDDSI [29]. This special property of smoothies was matched with the ability to hold food boluses in older individuals. Low-viscosity liquids like Ensure^®^ require thickening agents to enhance safety for individuals with dysphagia [34]. The second and third signs were retention/pooling of material and/or secretion, and the passage of food through the larynx or trachea. No significant differences were observed in the latter two signs between the formula diets in both the ASD and SSD groups. The highest severity was found when Ensure^®^ was swallowed in both the ASD and SSD groups. This study has several limitations that must be considered when interpreting the results. Firstly, the absence of long-term data restricts our understanding of the sustained effects of the interventions on nutritional status and health outcomes in older adults with dysphagia. Without extended follow-up, we cannot ascertain whether the observed benefits persist over time or if any delayed adverse effects emerge. Secondly, the use of non-validated swallowing screening questionnaires for group classification introduces potential biases. Non-validated tools may lack the necessary sensitivity and specificity, leading to misclassification of participants’ swallowing abilities. This misclassification could result in inaccurate assessments of the interventions’ efficacy and limit the generalizability of our findings. The European Society for Swallowing Disorders recommends discontinuing the use of non-validated dysphagia screening tools due to concerns about their diagnostic performance and psychometric properties [65]. These limitations underscore the necessity for future research employing validated diagnostic instruments and extended follow-up periods to evaluate the long-term efficacy and safety of such interventions accurately.

## Conclusion

Modified nutrition-dense smoothie diets were well-accepted by older adults and demonstrated improved flow characteristics before swallowing, outperforming less viscous foods like the comparator formula. These diets have the potential to enhance swallowing safety without the need for additional thickeners, addressing a critical challenge in managing dysphagia in older adults. The findings align with the need for safer, more acceptable food options for this population. To build on these findings, future research should focus on longitudinal studies to assess the long-term safety and efficacy of these formulas, as well as clinical trials to validate their impact on nutritional intake, swallowing function, and overall quality of life. Furthermore, the potential application of these diets in individuals with other chronic conditions or within diverse populations should be explored to further substantiate their broader relevance and contribute to the growing body of evidence on improving dietary interventions for vulnerable groups.

## Electronic supplementary material

Below is the link to the electronic supplementary material.

Supplementary Material 1

## Data Availability

The data used in the study is not publicly available, but the data used and/or analyzed during the current study are available from the corresponding author on reasonable request.

## Abbreviations

ASD: Asymptomatic swallowing difficulty
BSLC: Black sesame soy milk smoothie (low carbohydrate)
cP: Centipoise
CS: Chicken shitake smoothie
FEES: Fiberoptic Endoscopic Evaluation of Swallowing
IDDSI: International Dysphagia Diet Standardisation Initiative
NDD: National Dysphagia Diet
ONS: Oral nutrition supplements
SSD: Symptomatic swallowing difficulty
WS: White sesame soy milk smoothie
WSLC: White sesame soy milk smoothie (low carbohydrate)
