# Supplementary material for: Evaluating swallowing capacity in older adults with dysphagia: high protein, low carbohydrate smoothie formulas versus commercial formula
Source: BMC Geriatr. 2025 Jul 2;25:456. doi: 10.1186/s12877-025-06126-x (PMC12220473; doi:10.1186/s12877-025-06126-x)
Supplement: Supplementary file 1 — Supplementary Material 1 [file 12877_2025_6126_MOESM1_ESM.docx]

**Supplementary Appendix S1.**

**Development of modified nutrition-dense smoothie diets**

The development of modified nutrition-dense smoothie diets involved formulating four smoothies rich in macronutrients using locally available raw materials, enhanced with micronutrients from high-quality herbs and fruits. These formulations were tailored to suit the taste preferences of the Thai older population and adhered to the principles of balanced energy, targeting 1 kcal/ml. The primary ingredients included soy, eggs, vegetable oils, tropical fruits, sesame, and shiitake mushrooms. The formulation process entailed precise calculations and adjustments of nutrients for each recipe, emphasizing high protein content and low, particularly simple, carbohydrate levels. The ingredients were meticulously mixed and blended until achieving homogeneity. The final products underwent sterilization at a high standard (121°C) and were packaged in retort pouch containers, ensuring a one-year shelf life at room temperature in a dry environment. This collaborative effort with the Institute of Food Research and Product Development at Kasetsart University, Thailand, prioritized safety. Rigorous testing for microorganisms (*Salmonella* spp., *Escherichia coli*, *Staphylococcus aureus*, *Bacillus cereus*, *Clostridium* spp., and total coliforms), and other toxic substances (Tin, Lead, Mercury, Arsenic, Aflatoxin) were tested and revealed values within the normal range, aligning with the guidelines of the Thai Food and Drug Administration.

**Supplementary Appendix S2.**

**Screening questionnaires for separating participants into asymptomatic swallowing difficulty (ASD) and symptomatic swallowing difficulty (SSD) groups**

The screening questionnaire was developed based on both healthcare professionals’ experience and previous reports [1-4]. It was designed to check if individuals had experienced the following symptoms more than one time per week: 'Did you have a swallowing problem?'

- Food stuck in throat
- Choking on food
- Painful swallowing
- Throat irritation
- Nasal regurgitation
- Repeated swallowing
- Not able to eat dry foods or liquid foods

**References**

1. Dagna C, Avenali M, De Icco R, Gandolfi M, Solaro C, Restivo D, Bartolo M, Meneghello F, Sandrini G, Tassorelli C. From DYMUS to DYPARK: validation of a screening questionnaire for dysphagia in parkinson’s disease. Dysphagia. 2022:1-7.

2. Etges CL, Scheeren B, Gomes E, Barbosa LDR: Screening tools for dysphagia: a systematic review. In: Codas: 2014. SciELO Brasil: 343-9.

3. Kawashima K, Motohashi Y, Fujishima I. Prevalence of dysphagia among community-dwelling elderly individuals as estimated using a questionnaire for dysphagia screening. Dysphagia. 2004;19:266-71.

4. Simons JA, Fietzek UM, Waldmann A, Warnecke T, Schuster T, Ceballos-Baumann AO. Development and validation of a new screening questionnaire for dysphagia in early stages of parkinson's disease. Parkinsonism Relat Disord. 2014;20(9):992-8.

**A screening questionnaire**

1. Sex  Male  Female
2. Age _________ years
3. Underlying diseases (May select more than 1 item below)

- Hypertension
- Dyslipidemia
- Diabetes Mellitus
- Cancer
- Stroke
- Gastritis
- Others
- None

1. Types of foods that you were able to eat (May select more than 1 item below)

- Clear liquid foods
- Full-liquid foods
- Soft foods
- Semi-solid foods
- Viscous foods
- Solid foods
- Dry foods

1. Did you have a swallowing problem more than once per week? (May select more than 1 item below)

- Food stuck in throat
- Choking on food
- Painful swallowing
- Throat irritation
- Nasal regurgitation
- Repeated swallowing
- Not able to eat dry foods or liquid foods

**Supplementary Appendix S3.**

**A 9-point hedonic scale for sensory evaluation**

(1 = dislike extremely; 2 = very dislike; 3 = dislike moderately; 4 = dislike slightly; 5 = neutral; 6 = like slightly; 7 = like moderately; 8 = very like; 9 = like extremely)

**Pre-test**

| Sensory attributes | Scoring | | | | | | | | |
| --- | --- | --- | --- | --- | --- | --- | --- | --- | --- |
|  | 1 | 2 | 3 | 4 | 5 | 6 | 7 | 8 | 9 |
| Characteristic | ⬜ | ⬜ | ⬜ | ⬜ | ⬜ | ⬜ | ⬜ | ⬜ | ⬜ |
| Color | ⬜ | ⬜ | ⬜ | ⬜ | ⬜ | ⬜ | ⬜ | ⬜ | ⬜ |
| Smell | ⬜ | ⬜ | ⬜ | ⬜ | ⬜ | ⬜ | ⬜ | ⬜ | ⬜ |
| Viscosity | ⬜ | ⬜ | ⬜ | ⬜ | ⬜ | ⬜ | ⬜ | ⬜ | ⬜ |
| Satisfaction | ⬜ | ⬜ | ⬜ | ⬜ | ⬜ | ⬜ | ⬜ | ⬜ | ⬜ |

**Post-test**

| Sensory attributes | Scoring | | | | | | | | |
| --- | --- | --- | --- | --- | --- | --- | --- | --- | --- |
|  | 1 | 2 | 3 | 4 | 5 | 6 | 7 | 8 | 9 |
| Color | ⬜ | ⬜ | ⬜ | ⬜ | ⬜ | ⬜ | ⬜ | ⬜ | ⬜ |
| Smell | ⬜ | ⬜ | ⬜ | ⬜ | ⬜ | ⬜ | ⬜ | ⬜ | ⬜ |
| Taste | ⬜ | ⬜ | ⬜ | ⬜ | ⬜ | ⬜ | ⬜ | ⬜ | ⬜ |
| Viscosity | ⬜ | ⬜ | ⬜ | ⬜ | ⬜ | ⬜ | ⬜ | ⬜ | ⬜ |
| Homogeneity | ⬜ | ⬜ | ⬜ | ⬜ | ⬜ | ⬜ | ⬜ | ⬜ | ⬜ |
| Swallowing | ⬜ | ⬜ | ⬜ | ⬜ | ⬜ | ⬜ | ⬜ | ⬜ | ⬜ |
| Satisfaction | ⬜ | ⬜ | ⬜ | ⬜ | ⬜ | ⬜ | ⬜ | ⬜ | ⬜ |

**Supplementary Appendix S4.**

**The evaluation by healthcare professionals from the findings of the FEES**

The food residue was evaluated from the findings of the FEES for the presence or absence of post swallow residue in the four areas:

1) Retention of the bolus: food in the vallecular and/or pyriform regions of the pharynx,

2) Nasopharyngeal regurgitation: regurgitation of food or water into the nasal cavity,

3) Laryngeal penetration: food passing through the larynx (above the vocal cords)

4) Aspiration: food passing through the trachea (below the vocal cords)

The additional scores were also weighed toward three anatomical regions:

5) Premature spillage of material: the bolus leaks or falls into the hypopharynx before swallowing,

6) Retention/pooling of material and/or secretion: food or saliva residue in the vallecular and/or and/or pyriform regions of the pharynx after swallowing

7) Entrance of material and/or secretion into the larynx or trachea; presence/absence of reflex cough: food passes through the larynx or trachea when swallowed with or without the cough reflex

The healthcare professional rated the food residue in 5) and 6) as a percent of the space filled by assigning 5 scores based on the perception of the amount of residue compared to the total amount of bolus swallowed. A '5, none' represented no residue, '4, mild' refers to <26% remaining in the bolus, '3, moderate' refers to 26-50%, '2, marked' refers to 51-75% and “1, severe” referred to >75%.

The scores of 7) were classified according to the following criteria: '5' represented no entry of materials into the larynx or trachea, '4' referred to the entry of materials into the larynx above the trachea with reflex cough, '3' referred to the entry of materials into the larynx above the trachea without reflex cough, '2' referred to the entry of materials into the larynx with the formation of reflex cough, and '1' referred to the entrance of materials into the trachea without reflex cough.

**Fiber-optic Endoscopic Evaluation of Swallowing (FEES)**

| **Findings from FEES** | |
| --- | --- |
| Retention of bolus | ⬜ 0=not found ⬜ **1=found** |
| Nasopharyngeal regurgitation | ⬜ 0=not found ⬜ **1=found** |
| Laryngeal penetration | ⬜ 0=not found ⬜ **1=found** |
| Aspiration | ⬜ 0=not found ⬜ **1=found** |

| **Problem** | **Severity** | **Score** |
| --- | --- | --- |
| Premature spillage of material | ⬜ None: 0% total amount of bolus swallowed  ⬜ Mild: <26% total amount of bolus swallowed  ⬜ Moderate: 26-50% total amount of bolus swallowed  ⬜ Marked: 51-75% total amount of bolus swallowed  ⬜ Severe: >75% total amount of bolus swallowed | 5 scores  4 scores  3 scores  2 scores  1 score |
| Retention/pooling of material and/or secretion | ⬜ None: 0% total amount of bolus swallowed or saliva  ⬜ Mild: <26% total amount of bolus swallowed or saliva  ⬜ Moderate: 26-50% total amount of bolus swallowed or saliva  ⬜ Marked: 51-75% total amount of bolus swallowed or saliva  ⬜ Severe: >75% total amount of bolus swallowed or saliva | 5 scores  4 scores  3 scores  2 scores  1 score |
| Entrance of material and/or secretion into the larynx or trachea: presence/absence of reflex cough | ⬜ No entrance of materials into larynx or trachea  ⬜ Entrance of materials into larynx above trachea/reflex cough  ⬜ Entrance of materials into larynx about trachea/no reflex cough  ⬜ Entrance of materials into trachea with reflex cough forming  ⬜ Entrance of materials into trachea/no reflex cough | 5 scores  4 scores  3 scores  2 scores  1 score |
| **Total Score** | | …. / 15 scores |
| *Total score 15 scores means no swallowing difficulties | | |

**Supplementary Figure S1.** Test diets. Panel (A) The five types of diet for sensory tests. From left to right, these include WS, WSLC, BSLC, CS, and Ensure^®^ (control), Panel (B) The three types of diet for the swallowing test. From left to right, these include WS, CS, and Ensure^®^ (control), green-dyed water (for rinse after swallowing).


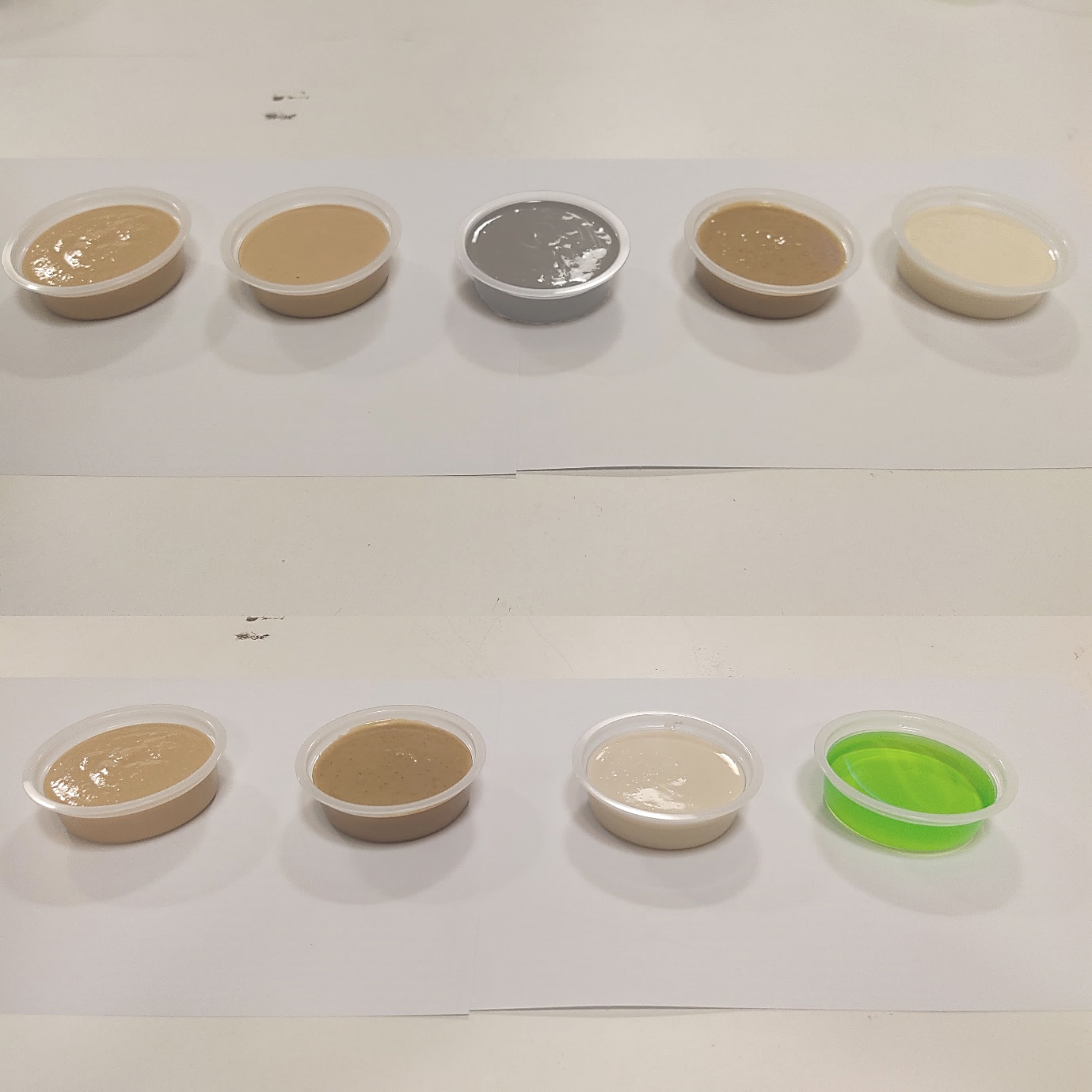

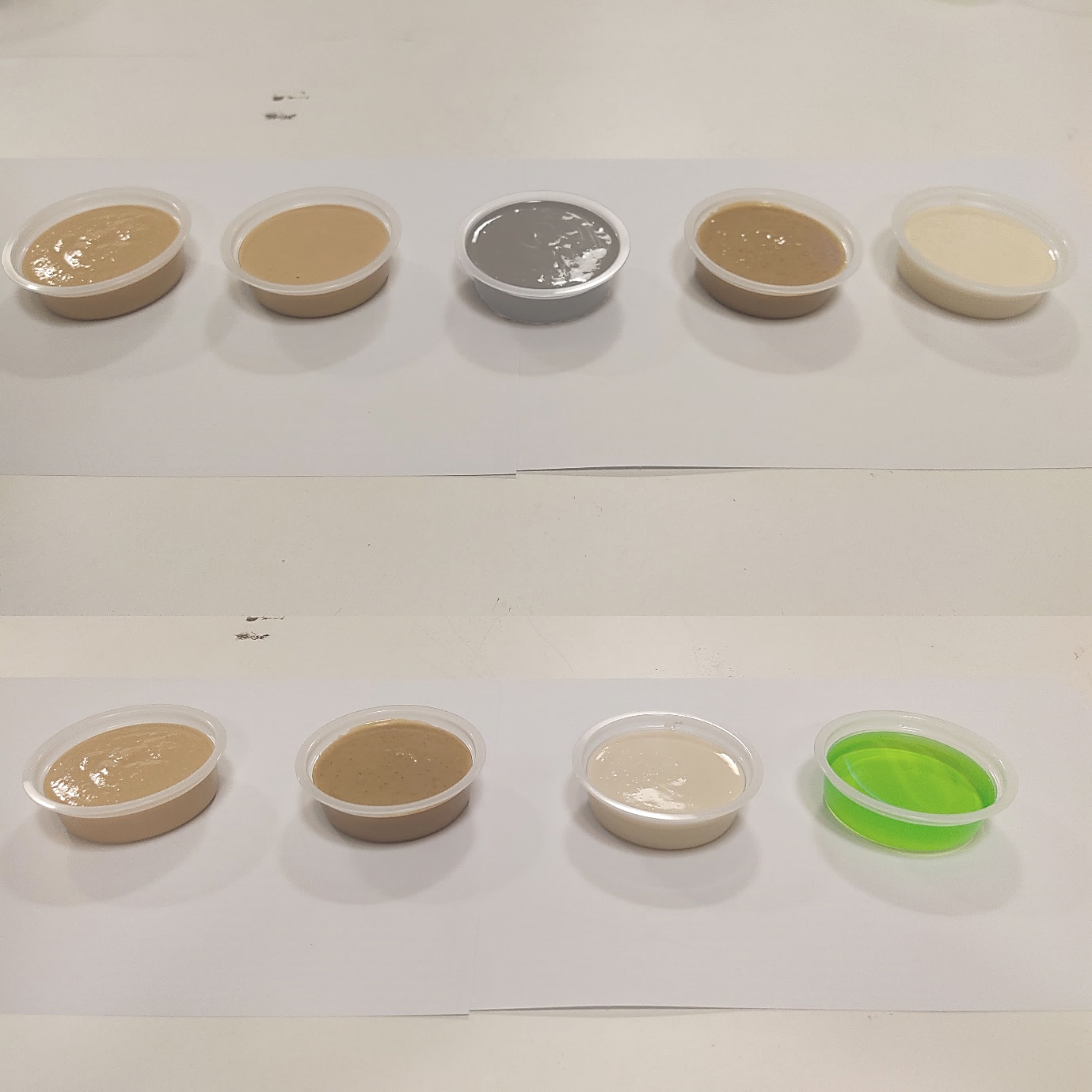


(A)

(B)

**Supplementary Table S1.** Sensory rating of key properties of test diets

| **Properties** | **WS** | | **WSLC** | | **BSLC** | | **CS** | | **Ensure^®^** | | ***p*-value*** |
| --- | --- | --- | --- | --- | --- | --- | --- | --- | --- | --- | --- |
|  | **Median** | **(IQR)** | **Median** | **(IQR)** | **Median** | **(IQR)** | **Median** | **(IQR)** | **Median** | **(IQR)** |  |
| **Panel 1:** Pre-test | | | | | | | | | | | |
| Characteristic | 5^a^ | (5-7) | 5^ab^ | (4-7) | 5^c^ | (3-5) | 5^b^ | (4-7) | 7^d^ | (5-8) | <0.001 |
| Color | 7^a^ | (5-8) | 6^ab^ | (5-7) | 5^c^ | (3-6) | 5^b^ | (5-7) | 7^d^ | (6-8) | <0.001 |
| Smell | 5^a^ | (5-7) | 5^a^ | (4-7) | 5^a^ | (5-7) | 5^a^ | (3-7) | 7^b^ | (7-8) | <0.001 |
| Viscosity | 6^ab^ | (5-7) | 5^b^ | (4-7) | 5^b^ | (3-7) | 7^ac^ | (5-7) | 7^c^ | (5-7) | 0.001 |
| Satisfaction | 6^a^ | (5-7) | 5^a^ | (5-7) | 5^a^ | (4-7) | 7^a^ | (5-7) | 7^b^ | (7-8) | <0.001 |
| **Panel 2:** Post-test | | | | | | | | | | | |
| Color | 7^a^ | (5-7) | 7^a^ | (5-7) | 5^b^ | (3-5) | 5^a^ | (5-7) | 8^c^ | (7-8) | <0.001 |
| Smell | 6^a^ | (5-7) | 5^ab^ | (4-7) | 5^b^ | (4-7) | 6^ab^ | (3-7) | 7^c^ | (7-8) | <0.001 |
| Taste | 7^a^ | (5-8) | 5^b^ | (4-7) | 5^b^ | (4-7) | 5^ab^ | (3-7) | 7^c^ | (6-8) | <0.001 |
| Viscosity | 7^a^ | (5-8) | 5^b^ | (4-7) | 5^b^ | (3-7) | 7^ab^ | (5-8) | 7^a^ | (5-8) | 0.001 |
| Homogeneity | 7^ab^ | (5-8) | 6^a^ | (4-7) | 6^a^ | (5-7) | 7^a^ | (5-8) | 7^b^ | (5-8) | 0.002 |
| Swallowing | 7^a^ | (5-8) | 6^a^ | (5-7) | 5^a^ | (4-7) | 7^a^ | (5-7) | 8^b^ | (6-8) | <0.001 |
| Satisfaction | 7^a^ | (5-8) | 6^a^ | (5-7) | 6^a^ | (4-7) | 7^a^ | (5-8) | 8^b^ | (7-8) | <0.001 |

^*^*p*-value for differences between five test meals using the nonparametric Kruskal-Wallis method (*p* < 0.05); Within a row, values with different superscript letters (a,b,c,d) are significantly different using Mann-Whitney comparisons (Bonferroni adjusted) (*p* < 0.05). Abbreviations: BSLC, black sesame soy milk smoothie (low carbohydrate); CS, chicken shitake smoothie; IQR, Interquartile range; WS, white sesame soy milk smoothie; WSLC, white sesame soy milk smoothie (low carbohydrate).

**Supplementary Table S2.** The findings of the FEES for the presence or absence of post-swallow residue in the four areas

| Test diets | Participant  group | Findings, n (%) | | | |
| --- | --- | --- | --- | --- | --- |
|  |  | Retention of bolus | Nasopharyngeal regurgitation | Laryngeal penetration | Aspiration |
| WS | ASD | 21 (65.6) | 0 (0.0) | 0 (0.0) | 0 (0.0) |
|  | SSD | 19 (61.3) | 0 (0.0) | 4 (12.9) | 0 (0.0) |
| CS | ASD | 15 (46.9) | 0 (0.0) | 0 (0.0) | 0 (0.0) |
|  | SSD | 19 (61.3) | 0 (0.0) | 2 (6.5) | 0 (0.0) |
| Ensure^®^ | ASD | 19 (59.4) | 0 (0.0) | 1 (3.1) | 0 (0.0) |
|  | SSD | 22 (71.0) | 0 (0.0) | 4 (12.9) | 0 (0.0) |

Abbreviations: ASD, asymptomatic swallowing difficulty; CS, chicken shitake smoothie; SSD, symptomatic swallowing difficulty; WS, white sesame soy milk smoothie.

**Supplementary Table S3.** The proportion of participants in each level of swallowing disorders by FEES

| **Participants, n (%)** | **ASD** | | | **SSD** | | |
| --- | --- | --- | --- | --- | --- | --- |
| **Test diets** | **WS** | **CS** | **Ensure^®^** | **WS** | **CS** | **Ensure^®^** |
| **(A) Premature spillage of material** | | | | | | |
| None | 31 (96.9) | 32 (100) | 27 (84.4) | 30 (96.8) | 29 (93.5) | 24 (77.4) |
| Mild | 1 (3.1) | 0 (0) | 4 (12.5) | 1 (3.2) | 2 (6.5) | 4 (12.9) |
| Moderate | 0 (0) | 0 (0) | 0 (0) | 0 (0) | 0 (0) | 3 (9.7) |
| Marked | 0 (0) | 0 (0) | 1 (3.1) | 0 (0) | 0 (0) | 0 (0) |
| Severe | 0 (0) | 0 (0) | 0 (0) | 0 (0) | 0 (0) | 0 (0) |
| ***p*-value^a^** | 0.105 | | | 0.068 | | |
| **(B) Retention/pooling of material and/or secretion** | | | | | | |
| None | 12 (37.5) | 17 (53.1) | 13 (40.6) | 12 (38.7) | 13 (41.9) | 9 (29.0) |
| Mild | 18 (56.3) | 13 (40.6) | 18 (56.3) | 10 (32.3) | 11 (35.5) | 17 (54.8) |
| Moderate | 1 (3.1) | 2 (6.3) | 0 (0) | 5 (16.1) | 3 (9.7) | 4 (12.9) |
| Marked | 1 (3.1) | 0 (0) | 1 (3.1) | 4 (12.9) | 3 (9.7) | 1 (3.2) |
| Severe | 0 (0) | 0 (0) | 0 (0) | 0 (0) | 1 (3.2) | 0 (0) |
| ***p*-value^a^** | 0.541 | | | 0.507 | | |
| **(C) Entrance of material and/or secretion into the larynx or trachea** | | | | | | |
| 5 scores | 32 (100) | 32 (100) | 31 (96.9) | 30 (96.8) | 30 (96.8) | 28 (90.3) |
| 4 scores | 0 (0) | 0 (0) | 1 (3.1) | 0 (0) | 0 (0) | 2 (6.5) |
| 3 scores | 0 (0) | 0 (0) | 0 (0) | 1 (3.2) | 0 (0) | 1 (3.2) |
| 2 scores | 0 (0) | 0 (0) | 0 (0) | 0 (0) | 0 (0) | 0 (0) |
| 1 score | 0 (0) | 0 (0) | 0 (0) | 0 (0) | 1 (3.2) | 0 (0) |
| ***p*-value^a^** | 0.364 | | | 0.313 | | |
| **Sum score of severity problem (A + B)** | | | | | | |
| 10 scores | 11 (34.4) | 17 (53.1) | 12 (37.5) | 12 (38.7) | 13 (41.9) | 9 (29.0) |
| <10 scores | 21 (65.6) | 15 (46.9) | 20 (62.5) | 19 (61.3) | 18 (58.1) | 22 (71.0) |
| ***p*-value^b^** | 0.265 | | | 0.547 | | |
| **Sum score of severity problem** (**A+B+C)** | | | | | | |
| 15 scores | 11 (34.4) | 17 (53.1) | 11 (34.4) | 12 (38.7) | 13 (41.9) | 9 (29.0) |
| <15 scores | 21 (65.6) | 15 (46.9) | 21 (65.6) | 19 (61.3) | 18 (58.1) | 22 (71.0) |
| ***p*-value^b^** | 0.211 | | | 0.547 | | |

^a^Fisher’s exact test. ^b^Chi-Square test. Abbreviations: ASD, asymptomatic swallowing difficulty; CS, chicken shitake smoothie; SSD, symptomatic swallowing difficulty; WS, white sesame soy milk smoothie.

**Supplementary Table S4.** Severity of swallowing disorders in test diets detected by FEES evaluation

| **Severity problem** | | **Premature spillage of material** | | **Retention/pooling of material and/or secretion** | | **Entrance of material and/or secretion into the larynx or trachea** | | **Sum score of severity problem** | |
| --- | --- | --- | --- | --- | --- | --- | --- | --- | --- |
|  |  | **Mean**  **(SEM)** | **Median**  **(IQR)** | **Mean (SEM)** | **Median (IQR)** | **Mean**  **(SEM)** | **Median**  **(IQR)** | **Mean**  **(SEM)** | **Median (IQR)** |
| ASD | WS | 5.0 (0.03) | 5 (5-5) | 4.3 (0.12) | 4 (4-5) | 5.0 (0.00) | 5 (5-5) | 14.3 (0.12) | 14 (14-15) |
|  | CS | 5.0 (0.00) | 5 (5-5) | 4.5 (0.11) | 5 (4-5) | 5.0 (0.00) | 5 (5-5) | 14.5 (0.11) | 15 (14-15) |
|  | Ensure^®^ | 4.8 (0.11) | 5 (5-5) | 4.3 (0.12) | 4 (4-5) | 5.0 (0.03) | 5 (5-5) | 14.1 (0.20) | 14 (14-15) |
|  | ***p*-value^a^** | 0.015 | | 0.116 | | 0.368 | | 0.035 | |
| SSD | WS | 5.0 (0.03) | 5 (5-5) | 4.0 (0.19) | 4 (3-5) | 4.9 (0.06) | 5 (5-5) | 13.9 (0.23) | 14 (13-15) |
|  | CS | 4.9 (0.04) | 5 (5-5) | 4.0 (0.20) | 4 (4-5) | 4.9 (0.13) | 5 (5-5) | 13.8 (0.28) | 14 (13-15) |
|  | Ensure^®^ | 4.7 (0.12) | 5 (5-5) | 4.1 (0.13) | 4 (4-5) | 4.9 (0.08) | 5 (5-5) | 13.6 (0.22) | 14 (13-15) |
|  | ***p*-value^a^** | 0.002 | | 0.565 | | 0.368 | | 0.444 | |

^a^Significance difference between test diets using Friedman test and pairwise Wilcoxon signed rank test. Abbreviations: ASD, asymptomatic swallowing difficulty; CS, chicken shitake smoothie; IQR, interquartile range; SEM, standard error mean; SSD, symptomatic swallowing difficulty; WS, white sesame soy milk smoothie.
